# Accounting for heterogeneity when estimating stopover duration, timing and population size of red knots along the Luannan Coast of Bohai Bay, China

**DOI:** 10.1002/ece3.5139

**Published:** 2019-06-18

**Authors:** Tamar Lok, Chris J. Hassell, Theunis Piersma, Roger Pradel, Olivier Gimenez

**Affiliations:** ^1^ CEFE, CNRS, Univ Montpellier, Univ Paul Valéry Montpellier 3, EPHE, IRD Montpellier France; ^2^ Department of Coastal Systems NIOZ Royal Netherlands Institute for Sea Research and Utrecht University Texel The Netherlands; ^3^ Global Flyway Network Broome Western Australia Australia; ^4^ Rudi Drent Chair in Global Flyway Ecology Conservation Ecology Group, Groningen Institute for Evolutionary Life Sciences (GELIFES) University of Groningen Groningen The Netherlands

**Keywords:** heterogeneity, Jolly–Seber, mark–recapture, migration, population size, red knot, state–space model, stopover duration

## Abstract

1. To successfully perform their long‐distance migrations, migratory birds require sites along their migratory routes to rest and refuel. Monitoring the use of so‐called stopover and staging sites provides insights into (a) the timing of migration and (b) the importance of a site for migratory bird populations. A recently developed Bayesian superpopulation model that integrates mark–recapture data and ring density data enabled the estimation of stopover timing, duration, and population size. Yet, this model did not account for heterogeneity in encounter (*p*) and staying (*ϕ*) probabilities.
2. Here we extended the integrated superpopulation model by implementing finite mixtures to account for heterogeneity in *p* and *ϕ*. We used simulations and real data (from 2009–2016) on red knots *Calidris canutus*, mostly of the subspecies *piersmai*, staging in Bohai Bay, China, during spring migration to (a) show the importance of accounting for heterogeneity in encounter and staying probabilities to get unbiased estimates of stopover timing, duration, and numbers of migratory birds at staging sites and (b) get accurate stopover parameter estimates for a migratory bird species at a key staging site that is threatened by habitat destruction.
3. Our simulations confirmed that heterogeneity in *p* affected stopover parameter estimates more than heterogeneity in *ϕ*, especially when most birds had low *p*. Bias was particularly severe when most birds had both low *ϕ* and *p*. Bias was largest for population size, intermediate for stopover duration and negligible for stopover timing.
4. A total of 50,000–100,000 red knots were estimated to annually stop for 5–9 days in Bohai Bay between 10 and 30 May. This shows the key importance of this staging site for this declining species. There were no clear changes in stopover parameters over time, although stopover population size was substantially lower in 2016 than in preceding years.
5. Our study shows the importance of accounting for heterogeneity in both encounter and staying probabilities for accurately estimating stopover duration and population size and provides an appropriate modeling framework.

## 1 INTRODUCTION

To successfully perform their seasonal long‐distance migrations, migratory birds require sites along the flyway to rest and refuel. Sites primarily used for resting are referred to as “stopover sites” (where individuals stay only briefly, i.e., for 1–2 days), whereas sites used for refueling are referred to as “staging sites” (Warnock, 2010). Duration of stay of migratory birds at such staging sites (hereafter called *stopover duration*) may range from several days up to several weeks. Habitat deterioration at staging sites has been shown to have major impact on the survival of migratory birds (Baker et al., 2004; Piersma et al., 2016) and may cause delays in migratory schedules and timing of arrival at the breeding grounds, with likely consequences for reproductive success (Daan, Dijkstra, Drent, & Meijer, 1989; Drent, Both, Green, Madsen, & Piersma, 2003; Smith & Moore, 2005). Monitoring stopover timing, duration, and population size of migratory birds at such staging sites is therefore important to detect changes in these parameters and guide conservation and management actions in order to better protect these sites and the migratory species that rely on them (Piersma & Baker, 2000).

While timing and duration of stopover can be directly measured for individuals with radio or satellite transmitters, in most cases, stopover duration is estimated from mark–recapture data of individually marked animals. To derive accurate estimates of timing and duration of stopover from marked individuals, it is important to account for imperfect detection, as individuals may have been present before their first observation and may have stayed for some time after their last observation (Kaiser, 1999). As a result, the time passed between the first and last observation of an individual (also known as “minimal stopover duration”) gives underestimated stopover durations (Kaiser, 1999; Schaub, Pradel, Jenni, & Lebreton, 2001) and the date of first observation is usually later than the true arrival date.

A flexible modeling framework to estimate timing and duration of stopover from individually marked birds is the superpopulation parameterization of the Jolly–Seber model (Jolly, 1965; Schwarz & Arnason, 1996; Seber, 1965). This model estimates entry and survival probabilities, while accounting for imperfect detection (by modeling encounter probabilities) and the fact that some individuals may have been missed entirely (i.e., especially those that stayed for a relatively short period). In the case of resightings of individually marked birds during migration at a staging site, entry and survival probabilities are interpreted as arrival and staying probabilities, assuming that mortality is negligible during the relatively short period of stopover (Lyons et al., 2016).

While estimates of timing and duration of stopover can be derived from encounters of individually marked birds at a staging site, additional information is required to estimate the total number of birds using a staging site. This can be achieved by capturing and marking new individuals at regular intervals at a staging site (Matechou, Morgan, Pledger, Collazo, & Lyons, 2013; Schwarz & Arnason, 1996), but this is usually impractical and may cause undesirable disturbance at a critical phase of the birds’ annual cycle. Another possibility is to perform daily counts of the total number of birds present at a site, which, when divided by stopover duration, gives the total number of birds using a site (Frederiksen, Fox, Madsen, & Colhoun, 2001). This method has the advantage that it is less time‐consuming and disturbing, but counting all birds present at a staging site, especially if the site is large and birds move around a lot, is prone to considerable errors. As an alternative, Lyons et al. (2016) recently developed an integrated model that combines observations of individually marked animals that have been marked away from the study site with scans of marked and unmarked individuals to estimate total stopover population size. In this model, the individual resighting data were analyzed using a Bayesian formulation of the Jolly–Seber model to estimate timing and duration of stopover as well as the number of marked individuals. To get from estimated number of marked birds to total number of birds (i.e., marked and unmarked birds combined), the Jolly–Seber model was combined with a binomial model that estimates the proportion of marked animals in the population from the scans of marked and unmarked birds.

The model of Lyons et al. (2016) assumes that individuals have equal encounter and staying probabilities. However, individuals may vary in their reliance on particular staging sites, with some individuals staying only for 1–2 days (e.g., while waiting for favorable weather conditions to continue migration), whereas others use the area for refueling and stay much longer. This will result in groups of birds with contrasting staying probabilities. In addition, birds may differ in space use within staging sites, causing some individuals to be more easily observed (because their feeding or roosting sites are better accessible to observers, e.g. close to the shore, or in publicly accessible areas as opposed to privately owned lands) than others, resulting in groups of birds with contrasting encounter probabilities.

Heterogeneity in encounter probabilities has already been shown to result in severe biases in population size estimates (Carothers, 1973). In the current paper, we extend the Bayesian model developed by Lyons et al. (2016) by implementing finite mixtures to account for individual heterogeneity, that is the presence of groups of individuals with contrasting encounter and staying probabilities. Finite mixtures enable the modeling of hidden classes of individuals with contrasting encounter and/or staying (or survival) probabilities and have previously been shown to adequately remove bias in parameter estimates of CJS models (Abadi, Botha, & Altwegg, 2013; Pledger, Pollock, & Norris, 2003) and Jolly–Seber models (Pledger, Pollock, & Norris, 2010) in the presence of individual heterogeneity. We use simulated and real data of red knots *Calidris canutus,* mostly of the subspecies *piersmai*, staging along the Luannan Coast of Bohai Bay, China, during spring migration to (a) show the importance of accounting for heterogeneity in encounter and staying probabilities to get unbiased estimates of stopover timing, duration, and numbers of migratory birds at staging sites and (b) get accurate estimates of stopover duration, timing, and population size for a migratory bird species at a key staging site that is threatened by habitat destruction (Piersma et al., 2016; Rogers et al., 2010), but that is currently listed to be conserved (Crockford, 2018).

## 2 MATERIAL AND METHODS

### 2.1 Study system

**Figure 1:**
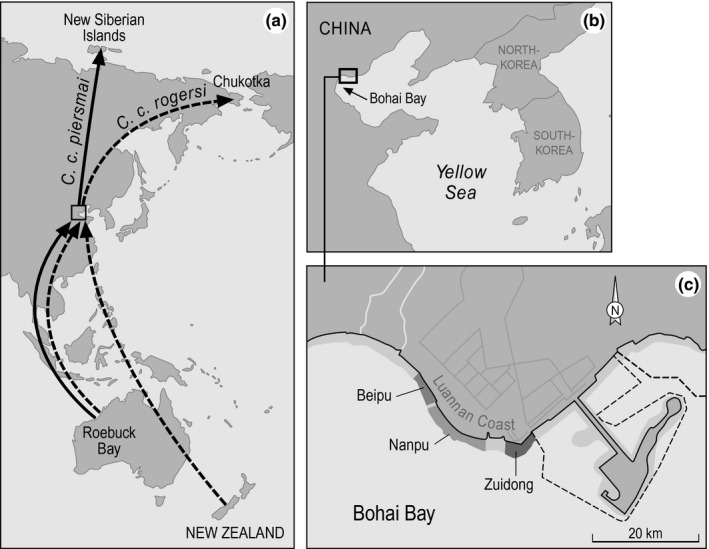
Maps of (a) the schematic northward migration routes of the red knot subspecies *C. c. piersmai and C. c. rogersi*, (b) the location of the study area in the Bohai Bay, and (c) the study area. Modified from Rogers et al. (2010)

Red knots that spend the winter in northwest Australia migrate along the East Asian–Australasian flyway to breed in the High Arctic (Figure 1; Piersma, 2007; Rogers et al., 2010). Between 2005 and 2016, 1,186 red knots *Calidris canutus,* mainly of subspecies *piersmai* but also of the morphologically distinct *rogersi* subspecies (Verhoeven, van Eerbeek, Hassell, & Piersma, 2016), were individually color‐banded at their nonbreeding grounds in Roebuck Bay and 80 Mile Beach, Northwest Australia (18–19°S, 120–122°E). Birds were caught using cannon nets, marked with a unique combination of color bands and a flag, and released within a few hours after capture (see Piersma et al., 2016 for details).

From 2009 onward, data were collected each year during the spring migration period on the presence of individually color‐marked red knots and on numbers of marked and unmarked birds at a key spring staging site, the Luannan Coast (covering the territories of the villages of Beipu, Nanpu and Zuidong) in northern Bohai Bay, China (39°N 118°E, Figure 1) (Yang et al., 2011). During this fieldwork, 2–4 observers visited the intertidal feeding areas and high tide roosts daily to collect the observations (see field reports available at www.globalflywaynetwork.com.au). Observers always carefully noted whether scans of the number of marked and unmarked birds performed on the same day involved different parts of the population (sampling without replacement) or whether scans were random samples of the population (sampling with replacement). Samples without replacement were merged into one large sample.

As years differed in the first and last date of fieldwork, we used data from 10 to 30 May to allow comparison between years and to have sufficient data to estimate daily arrival and encounter probabilities. Moreover, this is the core period of stopover of the subspecies *piersmai,* whereas the *rogersi* subspecies usually stops over earlier and longer (Rogers et al., 2010) and contains much fewer color‐banded individuals as this subspecies mainly winters in New Zealand (Figure 1). The number of different individuals of red knots observed per year during the selected period (10–30 May) ranged from 70 in 2009 to 312 in 2015. For a summary of the collected data, see Table 1 and Supporting Information Figures S1 and S2. Note that in 2010, no scans of numbers of marked and unmarked birds were performed.

**Table 1:** Data summary of yearly expeditions to Bohai Bay, shown, where applicable, for the period between 10 and 30 May (as used in the analysis) and for the entire study period (between brackets)

	2009	2010	2011	2012	2013	2014	2015	2016
Study period	11 May–29 May	15 April–28 May	17 April–30 May	16 April–3 June	14 April–4 June	10 April–5 June	16 April–6 June	14 April–4 June
Number of individuals seen	70 (70)	94 (109)	144 (155)	197 (248)	201 (245)	226 (299)	312 (365)	164 (242)
Mean number of daily observations per individual	1.6 (1.6)	1.7 (2.1)	1.9 (2.0)	2.4 (2.9)	1.8 (2.0)	2.2 (2.5)	2.4 (2.7)	1.8 (2.2)
Minimum stopover duration	3.2 (3.2)	3.6 (6.2)	4.8 (5.6)	5.1 (7.4)	3.2 (4.3)	4.8 (5.8)	5.1 (7.2)	4.2 (6.1)
Mean day in May of first observation	22 (22)	18 (13)	20 (18)	21 (19)	24 (22)	21 (20)	20 (18)	19 (15)

### 2.2 Jolly–Seber model to estimate migration parameters: assessing the presence of heterogeneity

We used open‐population Jolly–Seber models to estimate arrival (*β*), staying (*ϕ*), and encounter (*p*) probabilities, as well as number of marked red knots present at the study site per day (*M_t_*) and in total (M*). A key assumption of the Jolly–Seber model is homogeneity of *β*, *ϕ*, and *p* for marked and unmarked birds, which is critical for unbiased abundance estimation. Goodness‐of‐fit tests for the Jolly–Seber model are the same as those for the Cormack‐Jolly–Seber model (Pollock, Hines, & Nichols, 1985). We used the tests for CJS models to each year's data using R (R Core Team, 2015) and package R2ucare (Gimenez, Lebreton, Choquet, & Pradel, 2018). We separately show the results of Test3.SR and Test2.CT (as well as the overall test) as these tests tend to become significant when there is heterogeneity in, respectively, *ϕ* and *p*. The transience test (Test3.SR) was significant in 2014 only (*χ* ^2^ = 30.36, *df* = 16, *p* = 0.016), whereas the trap dependence test (Test2.CT) was significant in 2012 (*χ* ^2^ = 32.37, *df* = 15, *p* = 0.006) and 2015 (*χ* ^2^ = 55.84, *df* = 18, *p* < 0.001). An overview of all test results is listed in Supporting Information Table S1.

In addition to these tests, we investigated support for individual heterogeneity by comparing models with and without hidden classes of individuals (finite mixtures) with contrasting *ϕ* and *p*. To do so, we first explored support for daily variation in *ϕ* and *p* by comparing constant versus time‐dependent parameterizations using the computationally efficient frequentist formulation of the JSSA model as implemented in program MARK (the POPAN model). Arrival probabilities were modeled time‐dependent in all models, as we considered it biologically unrealistic that arrival probabilities would be the same at all days. In all years, the most parsimonious model (i.e., the model within ΔAIC_c_ < 2 with the fewest parameters) had constant *ϕ* and daily variation in *p* (*β_t_ϕ*_._ *p_t_*) (Supporting Information Table S2).

We then constructed the Jolly–Seber model in program E‐Surge (Choquet, Rouan, & Pradel, 2009) to enable the modeling of finite mixtures in the computationally efficient frequentist framework (Gimenez, Cam, & Gaillard, 2018). To implement open‐population models in E‐Surge, the option “conditional on first occasion” (instead of “conditional on first capture”) should be selected. For the model without mixtures, we defined three states: “not yet arrived,” “arrived,” and “departed.” The parameters were modeled as in the best‐supported model described above (*β_t_ϕp_t_*). To implement the finite‐mixture model with two classes of individuals, we defined four states: “not yet arrived,” “arrived in class 1,” “arrived in class 2,” and “departed.” We then compared models in which the two classes of individuals were assumed to vary (in an additive way) either or both in *ϕ* and *p* (*β_t_ϕ_h_p_t_*, *β_t_ϕ*_._ *p_t_*_+_ *_h_* and *β_t_ϕ_h_p_t_*_+_ *_h_*). As models were prone to local minima, each model was run five times with different initial values.

Models with two mixtures in *p* were supported in the years 2012–2015, with especially strong support in 2012 and 2015 (ΔAIC_c_ = 34.7 and 27.9) and considerable support in 2013 and 2014 (ΔAIC_c_ = 11.7 and 18.2) (Table 2). In 2012, there was additional support for heterogeneity in *ϕ* (Table 2).

**Table 2:** Model selection results of the JS‐model accounting for heterogeneity (*h*) in staying (*ϕ*) and/or encounter (*p*) probabilities by modeling two mixture classes, compared to the best‐supported model without heterogeneity, which for all years was the model with constant staying probability (*ϕ*_._) and daily variation in entry (*β_t_*) and encounter (*p_t_*) probabilities (Supporting Information Table S2)

Year	Model	*K*	ΔDev	ΔAIC_c_	Year	Model	*K*	ΔDev	ΔAIC_c_
2009	**β_t_ϕ_._p_t_**	**35**	**12.69**	**0.44**	2013	β_t_ϕ_._p_t_	40	18.89	11.63
	β_t_ϕ_._p_t+h_	37	12.27	9.19		**β_t_ϕ_._p_t+h_**	**42**	**2.14**	**0.00**
	β_t_ϕ_h_p_t_	37	3.09	0.00		β_t_ϕ_h_p_t_	42	11.09	8.95
	β_t_ϕ_h_p_t+h_	38	0.00	1.59		β_t_ϕ_h_p_t+h_	43	0.00	0.45
2010	**β_t_ϕ_._p_t_**	**40**	**6.67**	**0.00**	2014	β_t_ϕ_._p_t_	42	22.66	15.62
	β_t_ϕ_._p_t+h_	42	3.43	4.04		**β_t_ϕ_._p_t+h_**	**44**	**2.25**	**0.00**
	β_t_ϕ_h_p_t_	42	0.00	0.61		β_t_ϕ_h_p_t_	44	19.64	17.40
	β_t_ϕ_h_p_t+h_	43	3.00	7.34		β_t_ϕ_h_p_t+h_	45	0.00	0.17
2011	**β_t_ϕ_._p_t_**	**42**	**1.31**	**0.00**	2015	β_t_ϕ_._p_t_	43	36.86	27.90
	β_t_ϕ_._p_t+h_	44	0.05	4.41		**β_t_ϕ_._p_t+h_**	**45**	**3.95**	**1.77**
	β_t_ϕ_h_p_t_	44	0.00	4.36		β_t_ϕ_h_p_t_	45	15.62	13.44
	β_t_ϕ_h_p_t+h_	45	0.02	7.26		β_t_ϕ_h_p_t+h_	46	0.00	0.00
2012	β_t_ϕ_._p_t_	42	41.96	34.69	2016	**ϕ_._p_t_**	**43**	**6.45**	**0.00**
	β_t_ϕ_._p_t+h_	44	6.61	4.18		ϕ_._p_t+h_	45	1.48	0.53
	β_t_ϕ_h_p_t_	44	36.83	34.40		ϕ_h_p_t_	45	5.33	4.39
	**β_t_ϕ_h_p_t+h_**	**45**	**0.00**	**0.00**		ϕ_h_p_t+h_	46	0.00	1.85

Note

Each year's most parsimonious model is written in bold.

*K* = number of parameters; ΔDev = difference in deviance with the model with the lowest deviance of the same year; ΔAIC_c_ = difference in AIC_c_ with the model with the lowest AIC_c_ of the same year.

### 2.3 Bayesian formulation of finite‐mixture Jolly–Seber model combined with ring density model

To account for the individual heterogeneity in *ϕ* and *p* that we detected in our dataset, we refined and extended the Bayesian model developed by Lyons et al. (2016) to include finite mixtures. This model uses the hierarchical state–space formulation with data augmentation developed by Royle and Dorazio (2008) and implemented by Kéry and Schaub (2012). The state process is defined using latent state variable *z_i,t_* for individual *i* in the augmented dataset, where *z_i,t_* = 0 means that the individual has not yet arrived or has departed, whereas *z_i,t_* = 1 means that the individual is present at the site. Index *i* ranges from 1 to *A* (number of individuals in the augmented dataset) and index *t* from 1 to *K* (the number of sampling occasions, i.e., days). The inclusion of each individual in the augmented dataset is estimated by the latent variable *w_i_*, modeled as:

(1) $$w_{i}∼\text{Bernoulli}(\Psi )$$

where Ψ is the inclusion probability (being a function of the length of the augmented dataset, *A*). The total number of marked individuals in the population is then estimated as

(2) $${\hat{M}}^{*}=\sum_{i=1}^{A}w_{i}$$

We set *A* at three times the observed number of marked individuals. To verify that *A* was chosen sufficiently large (*A *>> M*), we visually verified that the posterior distribution of M* was not truncated (Kéry & Schaub, 2012).

The mixture class *h* that individual *i* belongs to is modeled as

(3) $$h_{i}∼\text{Categorical}\left(\Omega \right)$$

where Ω is a vector describing the discrete distribution of the mixture classes. The length of the vector reflects the number of mixture classes, which in our (particular) study case is set to two.

The state process is modeled as

(4) $$z_{i,t}|z_{i,t-1}∼\text{Bernoulli}\left(\phi_{t,h_{i}}z_{i,t-1}\right)$$

and the observation process as

(5) $$y_{i,t}|z_{i,t}∼\text{Bernoulli}\left(z_{i,t}p_{t,h_{i}}\right).$$

Stopover duration of each individual was derived from the latent state variables *z_i,t_* as

(6) $${\hat{S}}_{i}=\sum_{t=1}^{K}w_{i}z_{i,t}.$$

The mean stopover duration of the population was calculated as

(7) $$\hat{S}=\frac{\sum_{i=1}^{A}{\hat{S}}_{i}}{M^{*}}.$$

This calculation of mean stopover duration differs from Lyons et al. (2016). While Lyons et al. (2016) calculated mean stopover duration only over the individuals that were seen at least once, here, it is calculated over all individuals that were estimated to have stayed for at least one day, including those never observed. As a result, stopover duration estimates of the Lyons et al. (2016) model were positively biased, as never observed individuals were more likely to have stayed for a relatively short time.

The arrival probabilities (*β_t_*), reflecting the proportion of the overall population using the site that arrives at each day *t*, are modeled as in Lyons et al. (2016).

#### 2.3.1 Binomial model for ring density data

The proportion of marked individuals in the population ($\hat{\pi }$) is estimated from the ring density data (i.e., counts of marked and unmarked individuals) as

(8) $$m_{s}∼\text{Binomial}\left(N_{s},\hat{\pi }\right).$$

where *m*_s_ refers to the number of marked animals in the scan sample and *N*_s_ to the total number of animals (marked and unmarked) scanned. It is assumed that the proportion of marked individuals is constant over the season. The estimated proportion of marked individuals is then used to estimate the total population size (${\hat{N}}^{*}$) at the staging site:

(9) $${\hat{N}}^{*}={\hat{M}}^{*}/\hat{\pi }.$$

A key assumption of the Jolly–Seber model to produce unbiased parameter estimates, in particular of population size, is that all individuals (marked and unmarked) have the same entry, staying and encounter probabilities. By including mixtures in the model, we now account for heterogeneity in the form of two hidden groups that have distinct encounter and/or staying probabilities. With respect to the unmarked birds, it must now be assumed that the proportions of individuals in each mixture class are the same among marked and unmarked birds. This appears to be a reasonable assumption since the marking occurred at the wintering grounds; hence, the marked individuals have no (negative) association with certain areas or habitats at the staging site.

The JAGS code of the two‐mixture superpopulation model that accounts for heterogeneity in both *ϕ* and *p* is provided in Supporting Information Appendix S1.

The Bayesian integrated model was analyzed using Markov chain Monte Carlo simulations as implemented in JAGS (Plummer, 2003) via R (R Core Team, 2015) using R2jags (Su & Yajima, 2015). For each year of red knot data, two chains of length 30,000 were simulated and we used the last 20,000 iterations (excluding 10,000 burn‐in iterations) to describe the posterior distributions of the model (and derived) parameters. We used uninformative priors for all parameters. Convergence was assessed graphically and from the R‐hat values, assuming convergence when R‐hat values were smaller than 1.2 (Kéry & Royle, 2016). In addition, we checked that the Jolly–Seber model parameter estimates were similar when analyzed using MARK, E‐Surge, or JAGS (see Appendix S2 for an example of this check for the model *β_t_* *ϕ*_._ *p_t_* on the 2015 data).

### 2.4 Simulation study

We used simulations to assess the bias in model parameter estimates when heterogeneity in staying and encounter probabilities is ignored. To reflect the situation of the red knots in Bohai Bay, we assumed 15 occasions, 500 marked individuals, and two groups of individuals that differed in either or both encounter probability (*p* = 0.2 vs. *p* = 0.8) and staying probability (*ϕ* = 0.5 vs. *ϕ* = 0.9), with the proportion of individuals in the two groups being either low (0.2) or high (0.8). For the cases without heterogeneity, we assumed *p* = 0.5 and *ϕ* = 0.7. Arrival, staying, and encounter probabilities were assumed to be constant over time. This resulted in eight different scenarios, as summarized in Table 3. For each scenario, 1,000 mark–recapture datasets were simulated (*n*_sim_ = 1,000) and analyzed with the Jolly–Seber model in program MARK (White & Burnham, 1999) using R (R Core Team, 2015) and RMark (Laake, 2013). In addition, we analyzed 50 simulated datasets under scenario 1 and 5 of Table 3 with the Bayesian superpopulation model with two mixture classes to assess its performance in removing the bias. R‐code of the simulations is provided in Supporting Information Appendix S3.

Relative bias and associated mean square error (MSE) of parameter estimates are reported and calculated as follows:

$$\text{Relative}\;\text{bias}=\frac{1}{\theta n_{\text{sim}}}\sum_{i=1}^{n_{\text{sim}}}{\hat{\theta }}_{ı}-\theta$$

$$\text{MSE}=\frac{1}{\theta^{2}n_{\text{sim}}-1}\sum_{i=1}^{n_{\text{sim}}}{\left({\hat{\theta }}_{ı}-\theta \right)}^{2}$$

**Table 3:** Bias in Jolly–Seber model parameter estimates caused by heterogeneity in encounter (*p*) and/or staying (*ϕ*) probabilities

Scenario	Parameter	Simulated values			Model estimates			Relative bias	
		Per class		Population level	Mean	Confidence interval		Estimate	MSE
		Class 1	Class 2			2.5%	97.5%		
1–8	Ω	0.2	0.8						
1	*ϕ*	0.7	0.7	0.70	0.65	0.59	0.70	−0.074	0.007
	*P*	0.8	0.2	0.32	0.51	0.42	0.60	0.589	0.369
	*N*	100	400	500	359	312	410	−0.282	0.082
	Arrival day	8.00	8.00	8.00	8.06	7.00	9.00	0.007	0.005
	SOD	2.80	2.80	2.80	2.29	1.88	2.76	−0.175	0.037
2	*ϕ*	0.7	0.7	0.70	0.69	0.66	0.72	−0.016	0.001
	*p*	0.2	0.8	0.68	0.77	0.72	0.81	0.137	0.020
	*N*	100	400	500	453	433	474	−0.094	0.009
	Arrival day	8.00	8.00	8.00	8.01	7.00	9.00	0.002	0.003
	SOD	2.80	2.80	2.80	2.68	2.38	3.00	−0.039	0.005
3	*ϕ*	0.9	0.5	0.66	0.70	0.66	0.74	0.061	0.005
	*p*	0.5	0.5	0.50	0.47	0.41	0.52	−0.065	0.008
	*N*	100	400	500	484	445	529	−0.032	0.003
	Arrival day	8.00	8.00	8.00	8.03	7.00	9.00	0.004	0.005
	SOD	9.49	1.44	2.42	2.82	2.41	3.29	0.171	0.038
4	*ϕ*	0.5	0.9	0.87	0.87	0.86	0.89	0.009	0.000
	*p*	0.5	0.5	0.50	0.50	0.46	0.52	−0.006	0.001
	*N*	100	400	500	487	463	512	−0.027	0.001
	Arrival day	8.00	8.00	8.00	8.01	7.00	9.00	0.002	0.004
	SOD	1.44	9.49	6.93	7.37	6.37	8.75	0.070	0.013
5	*ϕ*	0.9	0.5	0.66	0.75	0.70	0.78	0.126	0.017
	*p*	0.8	0.2	0.49	0.74	0.69	0.79	0.507	0.260
	*N*	100	400	500	247	225	270	−0.505	0.256
	Arrival day	8.00	8.00	8.00	7.94	7.00	9.00	−0.008	0.005
	SOD	9.49	1.44	2.42	3.40	2.84	4.07	0.413	0.189
6	*ϕ*	0.5	0.9	0.87	0.89	0.87	0.90	0.024	0.001
	*p*	0.2	0.8	0.76	0.80	0.78	0.82	0.043	0.002
	*N*	100	400	500	435	422	447	−0.131	0.017
	Arrival day	8.00	8.00	8.00	8.00	7.00	9.00	0.000	0.003
	SOD	1.44	9.49	6.96	8.30	7.20	9.66	0.199	0.048
7	*ϕ*	0.9	0.5	0.66	0.61	0.55	0.66	−0.081	0.008
	*p*	0.2	0.8	0.50	0.56	0.47	0.66	0.102	0.019
	*N*	100	400	500	557	505	612	0.115	0.016
	Arrival day	8.00	8.00	8.00	8.15	7.00	9.00	0.019	0.004
	SOD	9.49	1.44	2.42	2.01	1.69	2.38	−0.168	0.033
8	*ϕ*	0.5	0.9	0.87	0.84	0.80	0.87	−0.033	0.002
	*p*	0.8	0.2	0.24	0.25	0.21	0.29	0.064	0.012
	*N*	100	400	500	544	491	607	0.088	0.011
	Arrival day	8.00	8.00	8.00	8.22	7.00	10.00	0.028	0.009
	SOD	1.44	9.49	6.95	5.59	4.41	7.15	−0.184	0.044
9 and 10	Ω	0.36	0.64						
9	*ϕ*	0.96	0.96	0.96	0.94	0.92	0.96	−0.020	0.001
	*p*	0.4	0.15	0.24	0.29	0.26	0.33	0.231	0.058
	*N*	180	320	500	449	416	487	−0.102	0.012
	Arrival day	8.00	8.00	8.00	8.17	7.00	9.00	0.022	0.008
	SOD	24.50	24.50	24.44	16.36	12.13	24.25	−0.310	0.113
10	*ϕ*	0.92	0.98	0.96	0.94	0.91	0.96	−0.027	0.001
	*p*	0.4	0.15	0.22	0.27	0.24	0.30	0.219	0.053
	*N*	180	320	500	475	435	516	−0.051	0.004
	Arrival day	8.00	8.00	8.00	8.17	7.00	9.00	0.021	0.008
	SOD	11.99	49.50	25.51	14.77	10.94	21.48	−0.403	0.175

Note

Ω = proportion in each mixture class; Arrival day = first day at which 50% of the population has arrived; *N* = superpopulation size; SOD = stopover duration (here calculated as −1/ln(*ϕ*)); MSE = mean square error.

## 3 RESULTS

### 3.1 Red knots in Bohai Bay

**Figure 2:**
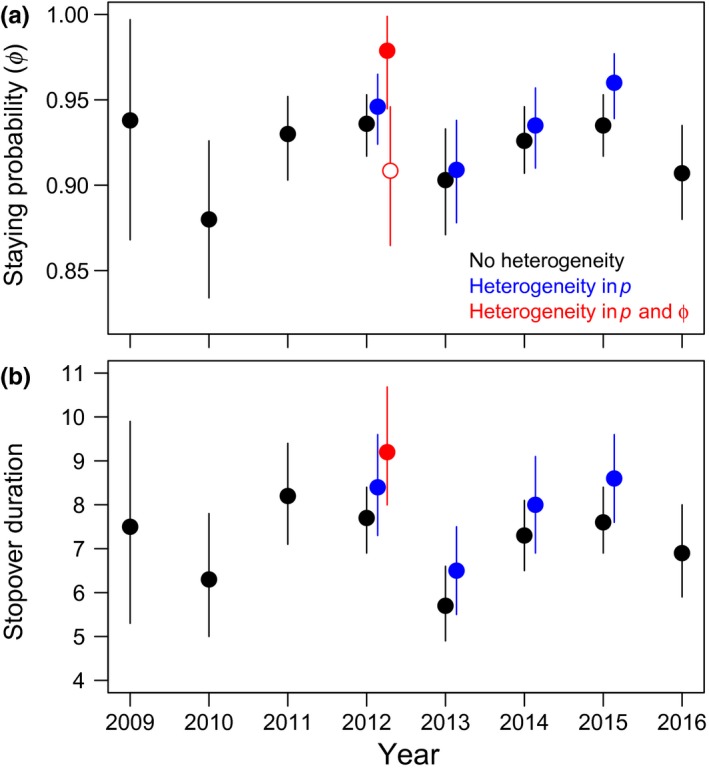
Estimated (a) staying probabilities and (b) stopover duration (in days) of models that did not account for heterogeneity (in black), and of two‐mixture models that accounted for heterogeneity in encounter probabilities (in blue) or heterogeneity in both encounter and staying probabilities (in red). The open and filled red dots in panel (a) represent the estimated staying probabilities of the two mixture classes, with an estimated 35% (21%–53%) of the population having relatively low staying probabilities in 2012. Posterior means and 95% credible intervals are shown

**Figure 3:**
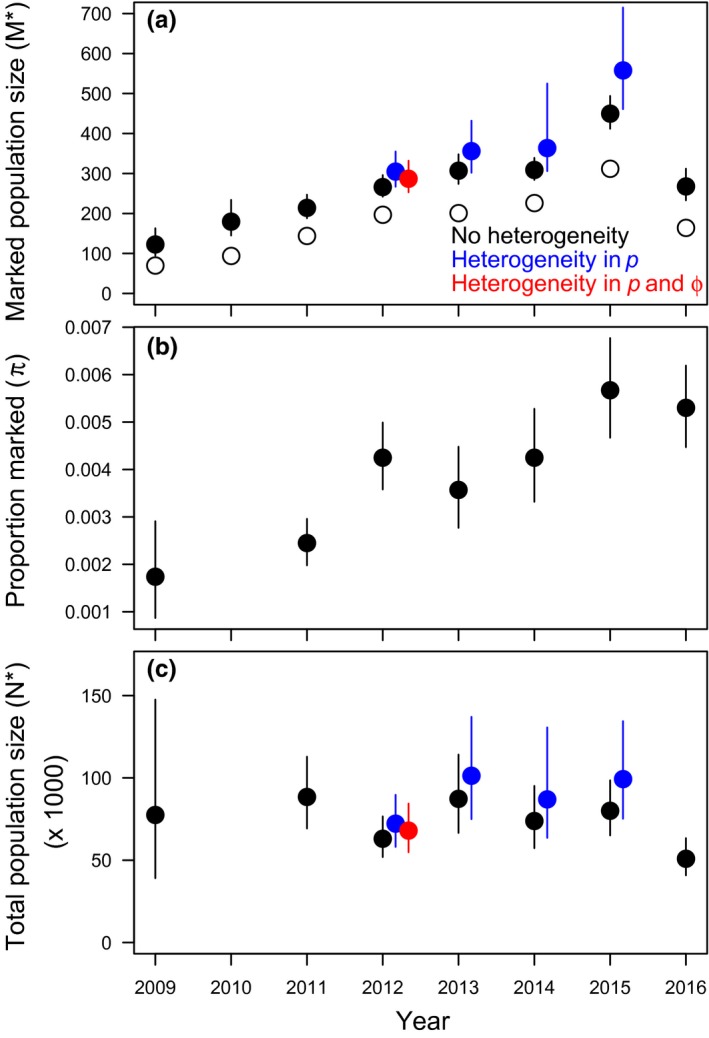
(a) The observed (open dots) and estimated (filled dots) number of marked individuals, (b) the estimated proportion of the population that is marked, and (c) the estimated total population size from the models that do not account for heterogeneity (in black) and the two‐mixture models that account for heterogeneity in encounter probabilities only (in blue) or for heterogeneity in both encounter and staying probabilities (in red). Posterior means and 95% credible intervals are shown. Because no ring density scans were performed in 2010, total stopover population size could not be estimated for this year

**Figure 4:**
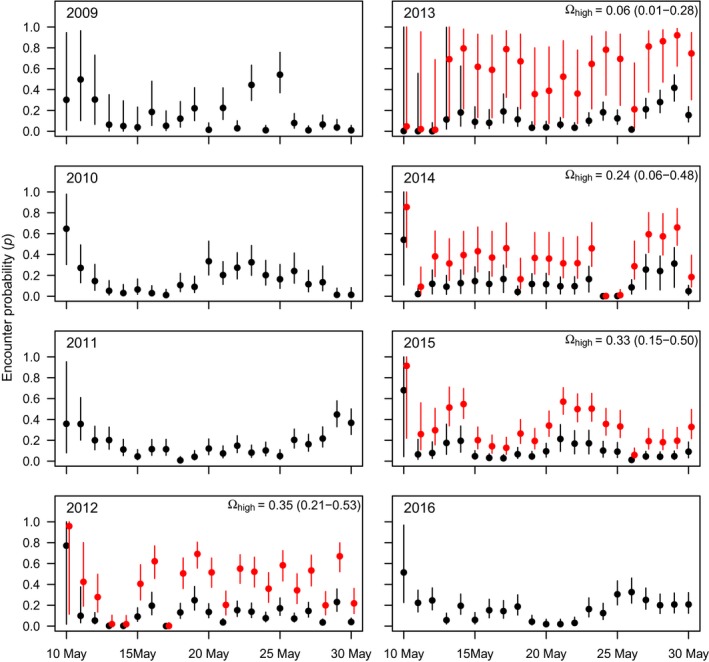
Estimates of encounter probabilities of the best‐supported models per year (*β_t_* *ϕ*_._ *p_t_* for 2009–2011 and 2016, *β_t_* *ϕ*_._ *p_t_*_+h_ for 2013–2015, and *β_t_* *ϕ*_h_ *p_t_*_+h_ for 2012). For the years with heterogeneity in *p* (2012–2015), estimates are plotted separately for the poorly resightable (in black) and highly resightable birds (in red), where $\Omega_{\text{high}}$ is the proportion of highly resightable individuals in the population. Posterior means and 95% credible intervals are shown

The Bayesian analysis of the integrated superpopulation model showed that in the years 2009 to 2016, a total of 50,000–100,000 red knots annually stopped along the Luannan Coast in Bohai Bay (China) for about 6 to 9 days between 10 and 30 May (Figures 2 and 3). We applied the two‐mixture model for the years with support for heterogeneity in *p* and/or *ϕ* (2012–2015, see Section 2). In these years, a small proportion of birds (0.06–0.35) was estimated to have relatively high encounter probabilities (Figure 4). Ignoring heterogeneity in *p* resulted in underestimated staying probabilities, stopover duration, and population size (Figures 2 and 3, black vs. blue dots). Assuming that the mixture models provided unbiased estimates, parameter estimates were negatively biased by 1%–3% for *ϕ*, 8%–12% for stopover duration and 13%–19% for stopover population size (calculated from the mean values in Figures 2 and 3).

In 2012, there was support for heterogeneity in both *p* and *ϕ*. In this year, the majority of birds (65%) had relatively high staying (Figure 2) and low encounter probabilities (Figure 4). Assuming that the two‐mixture model with heterogeneity in *p* and *ϕ* provided unbiased estimates, when ignored, this linked heterogeneity resulted in slightly underestimated population sizes (bias of −7%, Figure 3, black dots vs. red dots) but more strongly underestimated stopover duration (bias of −17%) compared to years with only heterogeneity in *p* (−8 to −12%, Figure 2).

Timing of stopover, calculated as the first day at which 50% of the population had arrived (assessed from the cumulative sum of the entry probabilities), was similar between years. Ignoring the heterogeneity in encounter and/or staying probabilities caused little bias in estimates of timing of stopover (Supporting Information Figure S3).

### 3.2 Simulation study

The simulation study showed that heterogeneity in *p* and *ϕ* caused negligible bias in estimated mean arrival dates (<2.8% for all scenarios, Table 3) and that the extent of bias in population size and stopover duration estimates strongly depended on the proportions of birds with low and high *p* and/or *ϕ*.

With heterogeneity in only *p* (scenarios 1 and 2 in Table 3), the scenario where 80% of the individuals had *p* = 0.2 and 20% had *p* = 0.8 resulted in the largest (negative) biases, with *ϕ* being underestimated by 7.4%, stopover duration (calculated as −1/ln(*ϕ*)) by 17.5% and population size by 28.2% (Table 3). When 80% instead of 20% of the individuals had *p* = 0.8, biases were much smaller, with *ϕ*, stopover duration, and population size being underestimated by 1.6%, 3.9%, and 9.4%, respectively.

Heterogeneity in staying probabilities alone (scenarios 3 and 4 in Table 3) caused only small bias of population size estimates (−3.2% and −2.7%), but the scenario where the minority of individuals had high staying probabilities (scenario 3) caused considerable overestimation (by 17.1%) of stopover duration.

Heterogeneity in both *p* and *ϕ* (scenarios 5 to 8, Table 3) caused most severe bias when the minority of birds had high encounter and staying probabilities (scenario 5, Table 3), with population size being underestimated by 50.5% and stopover duration being overestimated by 41.3%. In all four scenarios, stopover duration was considerably biased (in either direction), with absolute bias ranging from 16.8% to 41.3% (Table 3).

Analyzing the simulated datasets of scenarios 1 and 5 (i.e., the scenarios that caused the most severe biases when heterogeneity was ignored, see Table 3) with the Bayesian superpopulation model with two mixture classes strongly reduced the bias in estimates of population size and stopover duration to values below 12% (Supporting Information Table S3). This implied a reduction in bias of 60%–97% compared to the models that did not account for heterogeneity (Table 3).

## 4 DISCUSSION

### 4.1 General conclusions

In this study, we extended the Bayesian superpopulation model developed by Lyons et al. (2016) by accounting for heterogeneity in encounter and staying probabilities through finite mixtures in order to provide unbiased estimates of stopover timing, duration, and population size of migratory birds. Using a combination of analyses of simulated and real data, we showed that heterogeneity in encounter and staying probabilities is present in real datasets and can cause severely biased parameter estimates, especially of stopover duration and population size. Accounting for heterogeneity where needed, the model estimated that between 10 and 30 May, a total of 50,000–100,000 red knots used the Luannan Coast of Bohai Bay (China) for about a week (6 to 9 days), to refuel for their (presumably) final migratory flight to the New Siberian Islands.

### 4.2 Statistical considerations

Our findings are in agreement with previous studies that showed that heterogeneity in encounter probabilities can severely bias population size estimates (Carothers, 1973; Cubaynes et al., 2010; Pledger et al., 2010), and to a lesser extent survival probabilities (Abadi et al., 2013). Bias in parameter estimates was particularly severe when a small group of birds had high *p* (scenario 1, Table 3), and most severe when this same group also had relatively high *ϕ* (scenario 5, Table 3). Such linked heterogeneity in *p* and *ϕ* may be common in natural populations, when the highly detectable individuals are the most dominant or most active, hence potentially the better surviving (or longer staying) individuals. Such linked heterogeneity was indeed found in a study of wolves *Canis lupus* (Cubaynes et al., 2010).

Moreover, while bias of *ϕ* was relatively small for most simulated scenarios, its exponential relationship with stopover duration (or life expectancy) makes bias of stopover duration (or life span) estimates much larger, especially when staying (or survival) probabilities are high. This is similar to a small difference in survival probability resulting in a much larger difference in population growth rate, especially among long‐lived species (Fletcher et al., 2012).

The heterogeneity in *p* among red knots in the years 2012–2015, with the majority of birds having low encounter probabilities, comes closest to simulated scenario 1 in Table 3. Yet, the difference in encounter probabilities of red knots was less extreme (approximately 0.15 vs. 0.4) than in the simulation (0.2 vs. 0.8). This may explain why the bias of parameter estimates in the red knot case study was in the same direction, but smaller than in the simulated scenario. This was confirmed by a simulated scenario more similar to the red knot situation (scenario 9, Table 3). In 2012, individuals differed in both *p* and *ϕ*, with the majority having high staying and low encounter probabilities, similar to scenario 8 of Table 3. However, in contrast to scenario 8, where ignoring such heterogeneity resulted in an overestimation of population size, the population size of red knots in 2012 was (slightly) underestimated. This can be explained by the relatively small difference in staying probabilities of red knots (0.92 vs. 0.98) compared to the simulation (0.5 vs. 0.9). A simulation with parameter values more similar to the red knot case confirmed that in this situation, population size becomes negatively biased (scenario 10, Table 3).

Depending on the main interest, different parameterizations of the Jolly–Seber model can be used. We used the superpopulation parameterization that allows direct modeling of entry probabilities, thereby allowing these probabilities to be modeled as a (non)linear function (instead of daily variation). When, on the other hand, interest lies in age‐dependent staying probabilities (one could imagine that staying probabilities decrease with the time already spent at the site (Pledger, Efford, Pollock, Collazo, & Lyons, 2009)), the multistate parameterization (and implemented in E‐Surge) may be better suited, as it enables the state “arrived” to be divided into multiple states “arrived since 1 day”, “arrived since 2 days”, etc. (Pradel, 2009), subsequently allowing the effect of age to be constrained by some (linear) function. In both cases, mixtures can be implemented to account for heterogeneity in encounter and/or staying probabilities.

The yearly estimates of total number of red knots (Figure 3c) were rather imprecise, which is due to the very low proportion of marked birds *π* (0.002–0.006, Figure 3b). The lower *π* is, while the uncertainty around this estimated proportion (*SE*) stays the same, the more imprecise the total population size estimate becomes. In our study, there was evidence for a (slight) positive temporal trend in *π*. However, because of the relatively low number of birds scanned per day, adding an additional parameter to the binomial model to account for this temporal trend considerably decreased the precision of *π*.

### 4.3 Biological considerations

Our estimate of stopover population size of red knots stopping along the Luannan Coast reflects the part of the population that uses the site between 10 and 30 May. Most red knots staging during this period were assigned to the subspecies *piersmai* (Rogers et al., 2010 and Supporting Information Figure S1). While the majority of marked *piersmai* birds were observed for the first time after 10 May, some individuals were known to have arrived earlier, especially in the years 2012, 2015, and 2016 (Supporting Information Figure S1). Most of these birds presumably stayed for a longer time, as snow only starts to melt at the High Arctic breeding grounds of *piersmai* red knots around 10 June (Piersma et al., 2016), and the Luannan Coast is probably one of the final staging sites before the birds fly nonstop (over land) to the breeding grounds (Hua, Piersma, & Ma, 2013). However, we cannot exclude the possibility that some of these birds moved to a different site within the Yellow Sea area after visiting the Luannan Coast in early May. As such, the cut‐off at 10 May may have led to an underestimation of the total stopover population size of the *piersmai* subspecies of red knots and to an underestimation of their stopover duration, excluding part of the data of the long stayers that were present already before 10 May. Yet, due to the presence of large numbers of unmarked *rogersi* red knots in April and early May (Rogers et al., 2010), the proportion of marked birds during this early period of the spring migration season was very low (Supporting Information Figure S2) and the scanning effort insufficient to get precise estimates of this marked proportion to translate to meaningful estimates of stopover population size.

On the other hand, significant numbers of *piersmai* red knots were known to be still present after 30 May. Yet, to allow comparison among years, with expeditions terminated at 28–30 May in 2009–2011, we selected data until 30 May. We investigated the change in model estimates when analyzing the data until 6 June (for the years with available data after 30 May). While it hardly affected stopover duration, analyzing the data until 6 June resulted in somewhat higher stopover population sizes (Supporting Information Figure S4). This indicates that new birds are still arriving by the end of May and beginning of June.

Despite the large‐scale land reclamations and associated disappearance of shorebird foraging areas along the Yellow Sea (Murray, Clemens, Phinn, Possingham, & Fuller, 2014; Piersma et al., 2016), and an estimated population decline of red knots in this flyway (Studds et al., 2017), there are no obvious changes in stopover duration, timing, and numbers of red knots using Bohai Bay (Figures 2 and 3). One possibility is that red knots become more and more concentrated at the few remaining sites along the flyway, with the Luannan Coast being one of them, providing a super‐abundant food source (Yang et al., 2013), potentially enabling birds to persist in this area despite the disappearance of some of the foraging areas. If true, this implies that the proportion of the population relying on the Luannan Coast has increased over the years, a concentration process that may have started before the first year of this study, 2009 (Yang et al., 2011).

With the total population size of red knots (including both the *piersmai* and *rogersi* subspecies) being estimated at approximately 100,000 birds (Studds et al., 2017), of which about half are *piersmai* red knots, this implies that between 2009 and 2016, 50%–100% of the entire red knot population, and about the entire population of the *piersmai* subspecies, in this flyway relies on the mudflats and saltplans of the Luannan Coast for refueling their impressive long‐distance migrations. As such, this study shows the immediate priority of protecting this staging area to prevent further land reclamation to help conserve the red knots in this flyway.

## CONFLICT OF INTEREST

None declared.

## AUTHOR CONTRIBUTIONS

T.L. conceived the ideas and T.L., R.P., and O.G. developed the model. T.P. planned and raised the financial support for the color‐marking of red knots in Northwest Australia and the fieldwork in Bohai Bay, all carried out by field teams led by C.H. T.L. analyzed the data and led the writing of the manuscript. All authors contributed critically to the drafts and gave final approval for publication.

## Data Availability

Data are available from the Dryad Digital Repository https://doi.org/10.5061/dryad.dq7495s.
