# Supplementary material for: Accounting for heterogeneity when estimating stopover duration, timing and population size of red knots along the Luannan Coast of Bohai Bay, China
Source: Ecol Evol. 2019 Jun 18;9(11):6176–88. doi: 10.1002/ece3.5139 (PMC6580431; doi:10.1002/ece3.5139)
Supplement: Supplementary file 1 [file ECE3-9-6176-s001.docx]

## Supporting Information for

# Accounting for heterogeneity when estimating stopover duration, timing and population size of red knots along the Luannan Coast of Bohai Bay, China

Tamar Lok, Chris Hassell, Theunis Piersma, Roger Pradel & Olivier Gimenez

## Appendix S1.

JAGS code for the superpopulation model integrating mark-recapture and ring density data to estimate stopover timing, duration and population size in the presence of two hidden groups of individuals that differ in both staying and encounter probabilities.

model {

**# Priors and constraints**

for (i in 1:A){ # A is the number of individuals in the augmented

dataset

theta[i] ~ dcat(pi) # draws from a discrete distribution whether an

individual belongs to class 1 or 2

for (t in 1:(n.occasions-1)){

phi[i,t] <- phi.mean[theta[i]]

} #t

for (t in 1:n.occasions){

logit(p[i,t]) <- beta1[t] + beta2[theta[i]]

} #t

} #i

phi.mean[1] ~ dunif(0, 1) # Prior for mean survival of class 1

phi.mean[2] ~ dunif(0, 1) # Prior for mean survival of class 2

for (t in 1:n.occasions){

beta1[t] ~ dunif(-10,10) # beta1 gives the logit of the encounter

probabilities for mixture class 1.

} #t

**# Priors for the additive effect of mixture class 2 (mixture class 1 is the reference class)**

beta2[1] <- 0

beta2[2] ~ dunif(0, 10) # only positive values are allowed, to constrain

mixture class 1 to have lower encounter

probabilities, as an additive effect to the

temporal variation.

pi ~ ddirich(alpha) # Prior for probability to be in class 1 or 2 (where

pi[2]=1-pi[1]); alpha is provided as data = c(1,1)

psi ~ dunif(0, 1) # Prior for inclusion probability

**# Dirichlet prior for entry probabilities**

for (t in 1:n.occasions){

beta[t] ~ dgamma(1, 1)

b[t] <- beta[t] / sum(beta[1:n.occasions])

} #t

**# Convert entry probs to conditional entry probs**

eta[1] <- b[1]

for (t in 2:n.occasions){

eta[t] <- b[t] / (1-sum(b[1:(t-1)]))

} #t

**# Likelihood**

for (i in 1:M){

**# First occasion**

**# State process**

w[i] ~ dbern(psi) # Draw latent inclusion

z[i,1] ~ dbern(eta[1])

**# Observation process**

mu1[i] <- z[i,1] * p[i,1] * w[i]

y[i,1] ~ dbern(mu1[i])

**# Subsequent occasions**

for (t in 2:n.occasions){

**# State process**

q[i,t-1] <- 1 - z[i,t-1]

mu2[i,t] <- phi[i,t-1] * z[i,t-1] + eta[t] * prod(q[i,1:(t-1)])

z[i,t] ~ dbern(mu2[i,t])

**# Observation process**

mu3[i,t] <- z[i,t] * p[i,t] * w[i]

y[i,t] ~ dbern(mu3[i,t])

} #t

} #i

**# Binomial model for scan samples**

**# Priors**

pband ~ dbeta(1,1)

**# Likelihood**

for (i in 1:n){

m[i] ~ dbin(pband, K[i])

} #nscans

**# Calculate derived population parameters**

for (i in 1:A){

for (t in 1:n.occasions){

u[i,t] <- z[i,t]*w[i] # Deflated latent state (u)

} #t

} #i

for (i in 1:A){

recruit[i,1] <- u[i,1]

for (t in 2:n.occasions){

recruit[i,t] <- (1 - u[i,t-1]) * u[i,t]

} #t

} #i

for (t in 1:n.occasions){

Nband[t] <- sum(u[1:A,t]) # Banded pop size at time t

Nstop[t] <- sum(u[1:A,t])/pband # Stopover pop size at time t

Bband[t] <- sum(recruit[1:A,t]) # Number of banded entries at time t

Bstop[t] <- sum(recruit[1:A,t])/pband # Stopover pop entries at time t

for (j in 1:2) {

logit(psight[t,j]) <- beta1[t] + beta2[j]

} #j

} #t

Nsuperband <- sum(w[]) # Total marked superpop

Nsuperstop <- sum(w[])/pband # Total stopover superpop

for (i in 1:A) {

zstop[i] <- sum(u[i,1:n.occasions]) # individual stopover duration

} #i

zes <- sum(zstop[])/Nsuperband # mean stopover duration

}

## Appendix S2.

A comparison of parameter estimates and 95% confidence or credible intervals from MARK, E-Surge and JAGS of the model β_t_ ϕ_._ *p*_t_ for the year 2015.


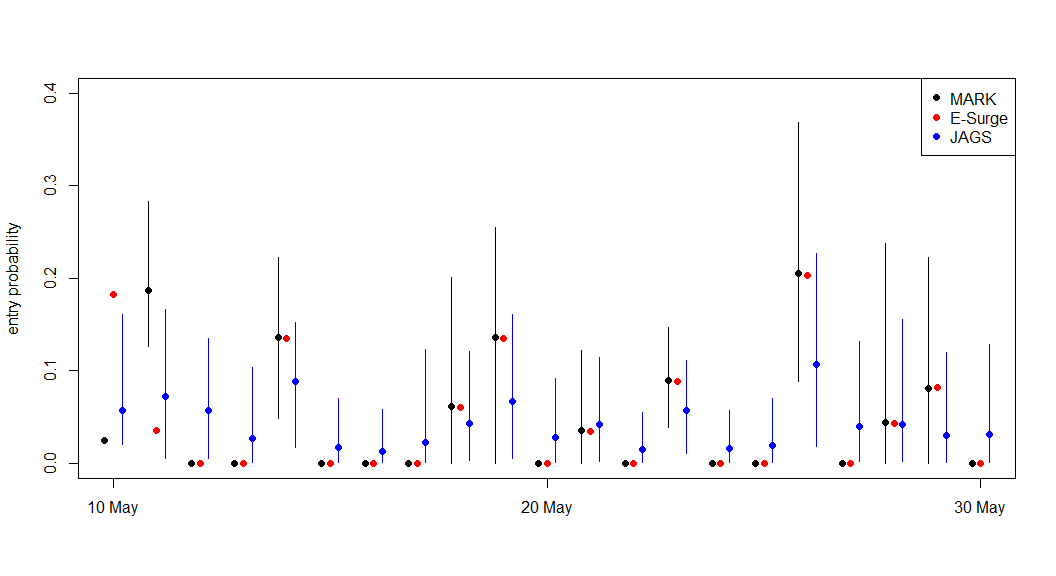


**Figure A2.1.** Entry probabilities (β). Note that the E-Surge estimates (in red) do not have confidence intervals, as these estimates are calculated from the conditional entry probabilities that are estimated in E-Surge. Estimates from MARK and E-Surge are virtually the same, while the mean estimates of JAGS are somewhat different. Yet, the credible intervals from JAGS overlap with the estimated means from MARK and E-Surge.


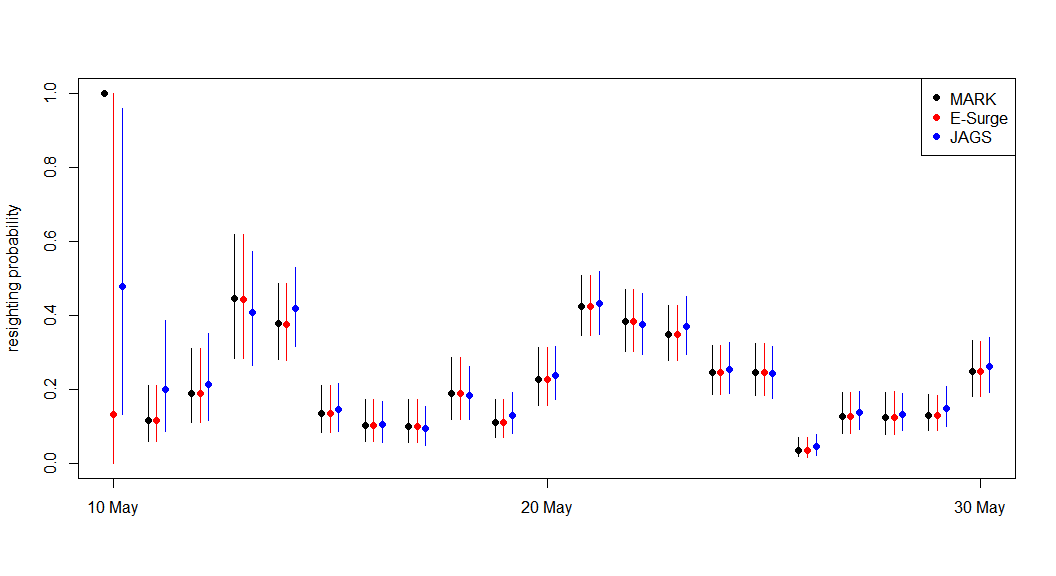


**Figure A2.2.** Resighting probabilities *p*.

**Table A2.1.** Staying probability ɸ and superpopulation size N.

|  | **MARK** | **E-Surge** | **Bayesian** |
| --- | --- | --- | --- |
| **ɸ** | 0.94 (0.92 - 0.95) | 0.94 (0.92 - 0.95) | 0.94 (0.92 - 0.95) |
| **N** | 455 (416 - 510) | - | 450 (412 - 494) |

## Appendix S3.

R-code for constructing and analysing simulated datasets. Code for simulating datasets conforming the Jolly-Seber model is adapted from Kéry & Schaub (2012) and Lyons *et al.* (2016).

library(RMark)

resample <- function(x, ...) x[sample.int(length(x), ...)]

**# Function to simulate capture-recapture data under the JS model**

simul.js <- function(PHI, P, b, N, pm, n.occasions) {

B <- rmultinom(1,N,b) # Generate no. of entering indiv. per occasion

CH.sur <- CH.p <- matrix(0, ncol=n.occasions, nrow=N)

**# Define a vector with the occasion of entering the pop**

ent.occ <- numeric()

for (t in 1:n.occasions) {

ent.occ <- c(ent.occ, rep(t, B[t]))

}

**# Simulate survival**

for (i in 1:N) {

CH.sur[i, ent.occ[i]] <- 1 # Write 1 when indiv. enters pop

if (ent.occ[i] == n.occasions) next # If arriving at last occasion, move to next individual

for (t in (ent.occ[i]+1):n.occasions) {

# Bernoulli trial: has indiv. survived occasion?

sur <- rbinom(1, 1, PHI[i,t-1])

ifelse (sur==1, CH.sur[i,t] <- 1, break) # If dead, move to next individual

} # t

} # i

**# Simulate capture**

for (i in 1:N) {

CH.p[i,] <- rbinom(n.occasions, 1, P[i,])

} # i

**# Full capture-recapture matrix**

CH <- CH.sur*CH.p

**# Assign marks (pm proportion of all individuals is marked)**

m <- rep(0, dim(CH)[1])

tagged <- sample(1:dim(CH)[1], pm*dim(CH)[1]) ## random selection of which birds were actually marked

m[tagged] <- 1

**# Remove individuals never seen (both marked and unmarked): removing individuals that were not seen after having assigned the marks, means that not exactly pm proportion of the individuals that were available for observation were marked.**

cap.sum <- rowSums(CH)

never <- which(cap.sum == 0)

CH.seen <- CH[-never,]

m.seen <- m[-never]

Nt <- colSums(CH.sur) # Population size (marked and unmarked

birds combined) at occasion t

CH.seen.marked <- CH.seen[m.seen==1,]

return(list(CH.seen.marked=CH.seen.marked, B=B, N=Nt, m.seen=m.seen, m=m, CH=CH, CH.sur=CH.sur, CH.p=CH.p))

}

**# Define general parameter values for all simulations**

Nsuper <- 10000 # Superpopulation size

pm <- 0.05 # Proportion marked

nsims <- 1000 # Number of simulations

n.occasions <- 15 # number of sampling occasions

b <- rep(1/n.occasions, n.occasions) # Entry probabilities

**# Define RMark formulas**

Phi.c = list(formula=~1)

p.c = list(formula=~1)

pent.time = list(formula=~time)

N.c = list(formula=~1)

**# Create function to construct and analyse a simulated dataset with specified parameters**

run.sim <- function(phi1, phi2=phi1, p1, p2=p1, prop1=0.2) {

N1 <- Nsuper * prop1 # Number of birds in the first class

N2 <- round(Nsuper * (1-prop1),0)

phi1 <- rep(phi1, n.occasions-1) # Staying probabilities of class 1

phi2 <- rep(phi2, n.occasions-1) # Staying probabilities of class 2

p1 <- rep(p1, n.occasions) # Encounter probability of class 1

p2 <- rep(p2, n.occasions) # Encounter probability of class 2

PHI1 <- matrix(rep(phi1, N1), ncol=n.occasions-1, nrow=N1, byrow=TRUE)

P1 <- matrix(rep(p1, N1), ncol=n.occasions, nrow=N1, byrow=TRUE)

PHI2 <- matrix(rep(phi2, N2), ncol=n.occasions-1, nrow=N2, byrow=TRUE)

P2 <- matrix(rep(p2, N2), ncol=n.occasions, nrow=N2, byrow=TRUE)

**# Make empty vectors and a list to store simulation results and data in**

Phi <- NA

N <- NA

p <- NA

pent <- matrix(NA, nrow=nsims, ncol=n.occasions-1)

sims <- list(NA)

Phi.true <- NA

p.true <- NA

SOD.true <- NA

**# Simulate datasets and analyse them with the POPAN model without mixtures**

for(j in 1:nsims) {

sim1 <- simul.js(PHI1, P1, b, N1, pm, n.occasions)

sim2 <- simul.js(PHI2, P2, b, N2, pm, n.occasions)

**# Extract presence data of marked individuals for the two groups**

present1 <- sim1$CH.sur[sim1$m==1,]

present2 <- sim2$CH.sur[sim2$m==1,]

present.all <- rbind(present1, present2)

**# Extract p data of marked individuals for the two groups**

seen1 <- sim1$CH.p[sim1$m==1,]

seen2 <- sim2$CH.p[sim2$m==1,]

seen.all <- rbind(seen1, seen2)

**# Calculate mean true Phi and SOD from presence data**

SODs <- rowSums(present.all)

SOD.true[j] <- mean(SODs)

survived <- NULL

seen <- NULL

**# Calculate true Phi and p from simulated dataset**

for (i in 1:(n.occasions-1)) {

survived <- c(survived, present.all[present.all[,i]==1,i+1]) # extract presence/absence vector at t=i+1 for individuals that were still present at t=i.

seen <- seen.all[present.all[,i]==1,i] # extract vector of individuals seen (1) or not seen (0) given that they were present

}

Phi.true[j] <- mean(survived)

p.true[j] <- mean(seen)

# extract the encounter history data for the two groups

CH1 <- sim1$CH.seen.marked

CH2 <- sim2$CH.seen.marked

**# Combine the two encounter history files:**

CH <- rbind(CH1, CH2)

ch <- data.frame(ch=apply(CH,1,paste,collapse=""), ind=1)

ch <- ch[as.numeric(ch$ch)>0,]

ch$ch <- as.character(ch$ch)

proc <- process.data(ch, model = "POPAN")

ddl <- make.design.data(proc)

model.sim <- mark(proc, ddl, model="POPAN", model.parameters = list(Phi=Phi.c, p=p.c, pent=pent.time, N=N.c), delete=T)

Phi[j] <- model.sim$results$real[1,'estimate']

p[j] <- model.sim$results$real[2,'estimate']

N[j] <- model.sim$results$real[3,'estimate']

pent[j,] <- model.sim$results$real[4:17,'estimate']

sims[[j]] <- CH

}

list(Phi=Phi, p=p, N=N, pent=pent, Phi.true=Phi.true, p.true=p.true, SOD.true=SOD.true, sims=sims)

}

## Table S1.

Results from U-Care goodness-of-fit tests for the red knot datasets for each year. The column Total shows the results summed over the four test components (Test3.SR, Test3.SM, Test2.CT and Test2.CI). P-values < 0.05 are indicated in bold.

| Year |  | Test3.SR: transience | Test2.CT:  trap dependence | Total |
| --- | --- | --- | --- | --- |
| 2009 | χ^2^ | 2.57 | 0 | 4.46 |
|  | df | 4 | 2 | 17 |
|  | p | 0.63 | 1 | 1 |
|  | c-hat | 0.64 | 0 | 0.26 |
| 2010 | χ^2^ | 7.61 | 8.84 | 23.75 |
|  | df | 9 | 9 | 37 |
|  | p | 0.57 | 0.45 | 0.96 |
|  | c-hat | 0.85 | 0.98 | 0.64 |
| 2011 | χ^2^ | 8.63 | 12.42 | 47.97 |
|  | df | 17 | 16 | 63 |
|  | p | 0.95 | 0.71 | 0.92 |
|  | c-hat | 0.51 | 0.78 | 0.76 |
| 2012 | χ^2^ | 16.04 | 32.37 | 105.96 |
|  | df | 15 | 15 | 60 |
|  | p | 0.38 | **0.0057** | **0.0002** |
|  | c-hat | 1.07 | 2.16 | 1.77 |
| 2013 | χ^2^ | 8.23 | 12.57 | 43.16 |
|  | df | 14 | 14 | 56 |
|  | p | 0.88 | 0.56 | 0.9 |
|  | c-hat | 0.59 | 0.90 | 0.77 |
| 2014 | χ^2^ | 30.36 | 20.97 | 94.51 |
|  | df | 16 | 16 | 67 |
|  | p | **0.016** | 0.18 | **0.015** |
|  | c-hat | 1.90 | 1.31 | 1.41 |
| 2015 | χ^2^ | 27.31 | 55.84 | 135.38 |
|  | df | 18 | 18 | 83 |
|  | p | 0.07 | **<0.0001** | **0.0002** |
|  | c-hat | 1.52 | 3.10 | 1.63 |
| 2016 | χ^2^ | 19.32 | 25.27 | 58.11 |
|  | df | 17 | 17 | 64 |
|  | p | 0.31 | 0.09 | 0.68 |
|  | c-hat | 1.14 | 1.49 | 0.91 |

## Table S2.

Model selection results of the JS-model with staying (ϕ) and/or encounter (*p*) probabilities modelled either as constant (.) or with daily variation (t). All models have daily variation in entry probabilities. Each year’s most parsimonious model is written in bold. Models have been run in MARK using RMark.

| Year | Model | K | ΔDeviance | ΔAIC_c_ |  | Year | Model | K | ΔDeviance | ΔAIC_c_ |
| --- | --- | --- | --- | --- | --- | --- | --- | --- | --- | --- |
| 2009 | ɸ_._p_._ | 23 | 180.92 | 134.71 |  | 2013 | ɸ_._p_._ | 23 | 103.75 | 52.96 |
|  | **ɸ_._p_t_** | **35** | **1.00** | **0** |  |  | **ɸ_._p_t_** | **40** | **9.75** | **0** |
|  | ɸ_t_p_._ | 42 | 117.90 | 150.76 |  |  | ɸ_t_p_._ | 42 | 78.65 | 74.02 |
|  | ɸ_t_p_t_ | 54 | 0 | 110.95 |  |  | ɸ_t_p_t_ | 59 | 0 | 41.66 |
|  |  |  |  |  |  |  |  |  |  |  |
| 2010 | ɸ_._p_._ | 23 | 100.14 | 35.57 |  | 2014 | ɸ_._p_._ | 23 | 222.85 | 165.02 |
|  | **ɸ_._p_t_** | **40** | **11.29** | **0** |  |  | **ɸ_._p_t_** | **42** | **14.26** | **0** |
|  | ɸ_t_p_._ | 42 | 55.60 | 51.59 |  |  | ɸ_t_p_._ | 42 | 173.13 | 158.87 |
|  | ɸ_t_p_t_ | 59 | 0 | 69.47 |  |  | ɸ_t_p_t_ | 61 | 0 | 33.08 |
|  |  |  |  |  |  |  |  |  |  |  |
| 2011 | ɸ_._p_._ | 23 | 115.85 | 58.15 |  | 2015 | ɸ_._p_._ | 23 | 211.80 | 140.94 |
|  | **ɸ_._p_t_** | **42** | **8.38** | **0** |  |  | **ɸ_._p_t_** | **43** | **26.97** | **0** |
|  | ɸ_t_p_._ | 42 | 84.23 | 75.86 |  |  | ɸ_t_p_._ | 42 | 162.84 | 133.61 |
|  | ɸ_t_p_t_ | 61 | 0 | 49.87 |  |  | ɸ_t_p_t_ | 62 | 0 | 17.12 |
|  |  |  |  |  |  |  |  |  |  |  |
| 2012 | ɸ_._p_._ | 23 | 202.46 | 149.41 |  | 2016 | ɸ_._p_._ | 23 | 98.98 | 35.04 |
|  | **ɸ_._p_t_** | **42** | **9.22** | **0** |  |  | **ɸ_._p_t_** | **43** | **13.20** | **0** |
|  | ɸ_t_p_._ | 42 | 158.42 | 149.21 |  |  | ɸ_t_p_._ | 42 | 65.36 | 49.43 |
|  | ɸ_t_p_t_ | 61 | 0 | 38.59 |  |  | ɸ_t_p_t_ | 62 | 0 | 42.9 |

## Table S3.

Analysis of 50 simulated datasets under scenario 1 and 5 (the two scenarios in Table 1 of the manuscript that resulted in the largest bias in parameter estimates) using the Bayesian superpopulation model with two mixture classes. In scenario 1, individuals all have the same staying probability (ϕ=0.7), but differ in resighting probability *p*, where 80% has *p=*0.2 and 20% has *p*=0.8. In scenario 5, individuals differed in both ϕ and *p*, where 80% of the population has *p=*0.2 and ϕ=0.5 and 20% has *p=*0.8 and ϕ=0.9. Simulated SOD (StopOver Duration) is calculated from the simulated state matrices, whereas the estimated SOD is calculated from the latent state variables estimated by the Bayesian model.

| Scenario | Parameter |  | Value | |  | Relative bias | |
| --- | --- | --- | --- | --- | --- | --- | --- |
|  |  |  | Simulated | Estimated |  | Estimate | MSE |
| 1 | N |  | 500 | 556 |  | 0.112 | 0.040 |
|  | Arrival day |  | 8.00 | 7.85 |  | -0.019 | 0.013 |
|  | SOD |  | 2.82 | 2.80 |  | -0.007 | 0.005 |
| 5 | N |  | 500 | 493 |  | -0.014 | 0.054 |
|  | Arrival day |  | 8 | 7.84 |  | -0.020 | 0.008 |
|  | SOD |  | 2.55 | 2.65 |  | 0.042 | 0.013 |


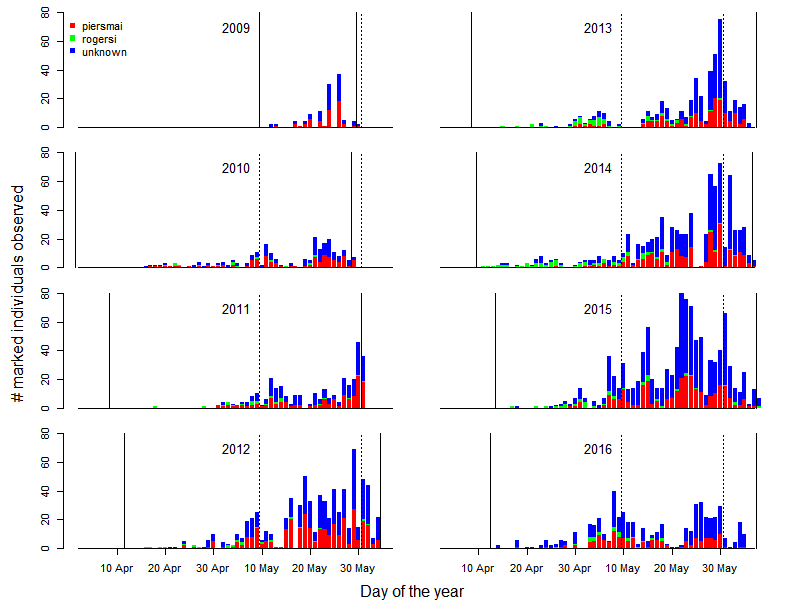


## Figure S1.

Number of marked individuals per subspecies observed per day in each year. The solid vertical lines indicate the start and end of the field season, whereas the dotted vertical lines indicate the period used for the analysis. Subspecies assignment was done on the basis of breeding plumage characteristics, and only for the males as their breeding plumage differs more clearly between subspecies than that of females.


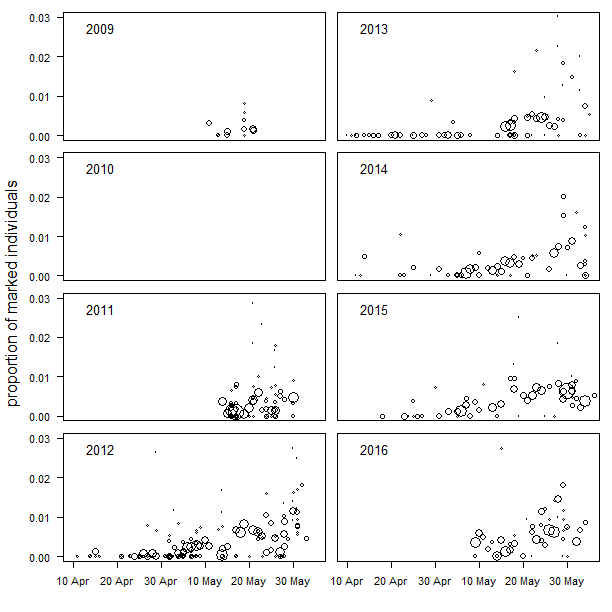


## Figure S2.

Proportion of marked individuals in counts of marked and unmarked individuals as a function of day of the year. The size of the dots reflects the number of birds scanned.


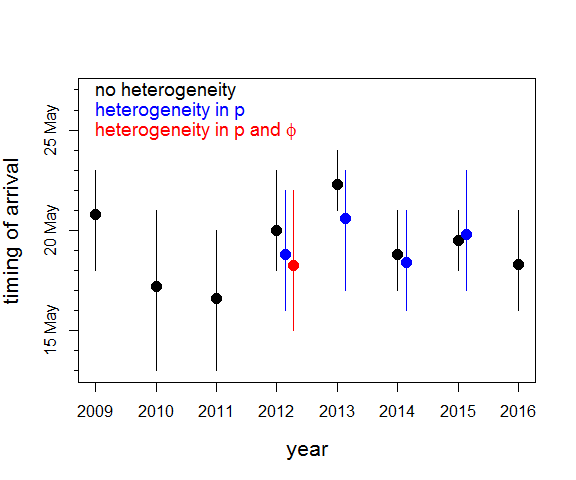


## Figure S3.

Estimated date of arrival, defined as the first date at which 50% of the population has arrived (calculated from the cumulative sum of the entry probabilities β*_t_*) from the models that do not account for heterogeneity (in black) and the two-mixture models that account for heterogeneity in encounter probabilities only (in blue) or for heterogeneity in both encounter and staying probabilities (in red). Posterior means and 95% credible intervals are shown.


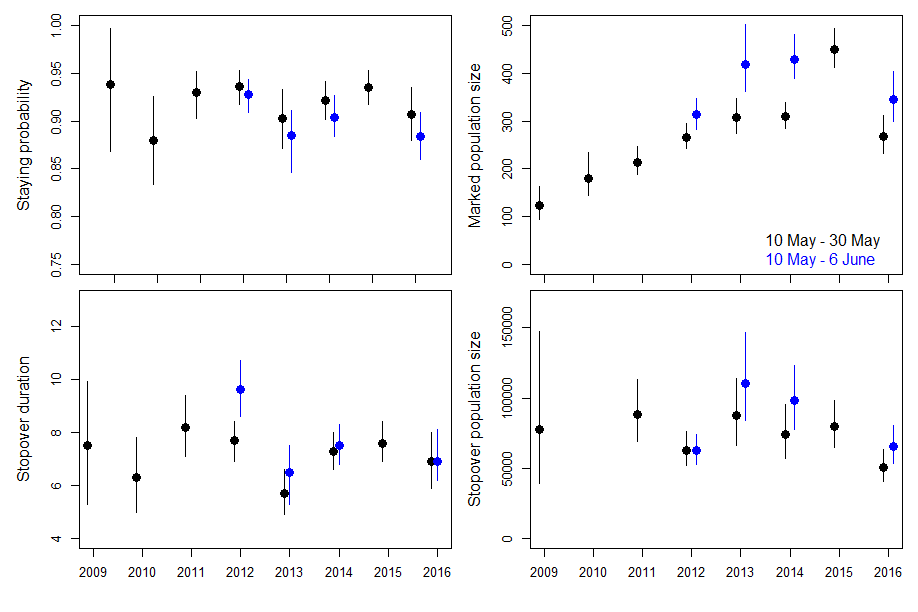


## Figure S4.

(a) Stopover duration, (b) staying probabilities and (c) marked population size and (d) stopover population size estimates of the model β_t_ ϕ_._ p_t_, for the datasets of 10 May - 30 May and 10 May - 6 June (only data until 4-6 June in the years 2012-2016). Stopover duration estimates do not differ much when an additional week of data is used, except in 2012. Marked and stopover population size estimates were higher in 2013 and 2014 when data until 6 June were used, indicating that in these years, new birds still arrived at the staging site after 30 May.

# References

Kéry, M. & Schaub, M. (2012) *Bayesian population analysis using WinBUGS: a hierarchical perspective*. Academic Press, Waltham, Massachusetts.

Lyons, J.E., Kendall, W.L., Royle, J.A., Converse, S.J., Andres, B.A. & Buchanan, J.B. (2016) Population size and stopover duration estimation using mark–resight data and Bayesian analysis of a superpopulation model. *Biometrics,* **72,** 262-271.
